# Comparison of the Effects of Video Modeling and Clinician Modeling on Naming Deficiency in Aphasic Patients

**DOI:** 10.31661/gmj.v0i0.1158

**Published:** 2019-01-01

**Authors:** Shohre Kaviani, Fatemeh Kasbi, Afshin Samaei, Ehsan Shahverdi

**Affiliations:** ^1^Neuromuscular Rehabilitation Research Center, Semnan University of Medical Sciences, Semnan, Iran; ^2^Neuromuscular Rehabilitation Research Centre, Department of Internal Medicine, Semnan University of Medical Sciences, Semnan, Iran; ^3^Department of Transfusion Medicine, Institute of Immunology and Transfusion Medicine, University Medicine Greifswald, Greifswald, Germany

**Keywords:** Aphasia, Video Modeling, Clinician Modeling, Naming Skills

## Abstract

**Background:**

Aphasia is the most frequent disorder that could occur following a stroke. Aphasia has a negative impact on the patient’s communication ability through language. One of the common consequences of aphasia is naming deficits that can lead to communication disorders. Therefore, the treatment of aphasia is necessary. The aim of the current study was to investigate the effect of video modeling and clinician modeling on naming skills of patients with chronic aphasia.

**Materials and Methods:**

The design of this prospective single subject study was ABA that performed on four patients with chronic aphasia. participated. This study was administered during three phases including the baseline (three sessions); the intervention (nine sessions); and a follow-up phase (three sessions). The outcome measure was taken in three phases including baseline, intervention, and follow-up. For each patient, the naming score for items modeled by the clinician, the naming score for items modeled video modeling by other, the naming score for self-video modeling, and the reaction time score were recorded.

**Results:**

A total of three patients complete the study and one of them refused to continue treatment. The naming score of all modeling types increased in all patients. In the other words, the intervention helped the patients be improved in naming. Also, the results of the reaction time indicated that the video modeling, as well as clinician modeling, could decrease the response time that means the intervention could increase the speed of retrieval processes.

**Conclusion:**

In our study, all three types of modeling could improve the naming scores in patients with chronic aphasia. Additionally, the findings demonstrate that the clinician and video modeling might increase mental processing for naming verbally.

## Introduction

At the first time, Bandura introduced the concept of observational learning as follows: “child’s ability in imitation or generalization can be improved through observing various skills or behaviors” [1, 2]. Video modeling by other and video self-modeling are two types of observational learning that participants watching the skills or behaviors modeled by adults, siblings, peers, or the individuals themselves through video representation [3]. Video modeling has been used to teach a wide range of skills or behaviors in various populations [4-10]. Video modeling has been used to improve different speech and language disorders such as Stuttering, Autism spectrum disorders, etc.[11]. However, there are a few studies about the efficiency of video modeling in treating acquired speech and language disorders such as aphasia. Indeed, aphasia is an acquired neurological disorder due to a stroke that can lead to multimodal language impairments [12]. Because of the high prevalence of aphasia following to stroke, and consequently naming deficits that lead to communication failure, the aim of this study was to investigate the efficacy of video modeling and clinician modeling on the naming skill in the patients with chronic aphasia.

## Materials and Methods

### 1. Study Design

The current study was a prospective, single-subject, A-B-A design, and case series study. We used Dryer’s model as a general template with some modifications according to our language, culture, and communication needs of patients [11].

### 2. Patients Selection

Four patients with first ischemic infarct within the left middle cerebral artery were recruited at the speech-language clinics. The inclusion criteria included the right-handed patients‒whom the Persian language was their first language‒, normal or corrected vision and hearing, relatively preserved auditory comprehension (at least 25 scores on the receptive index of Mississippi screening aphasia test[MAST]), and at least 12 months after stroke. The patients who encountered more than one cerebrovascular accidents, neurodegenerative, psychiatric disease, and epilepsy excluded from the study.Assessment, diagnosis, and intervention of aphasia in the current study were performed by the speech and language pathologist (SLP). In order to select the patients, after completing consent forms, they were screened by MAST [13] to diagnose the aphasia and the Nilipour naming test [14] was used to examining naming deficits. Also, the semantic knowledge of each participant was determined by naming test [15].

#### 2.1. Patient 1

A 53-year-old male who had a left frontotemporoparietal ischemic cerebrovascular accident (CVA) three years ago. He was diagnosed with moderate to severe Broca’s aphasia and apraxia of speech, according to speech and language evaluation. The receptive and expressive scores of MAST were 30 and 10, respectively. The score of Nilipour naming test was 20 that means he had a severe naming deficit. The semantic knowledge was measured by a naming test [15] and the results indicated raw score 298 out of 300. Also, he worked as a teacher.

#### 2.2. Patient 2

The second patient was a 49-year-old female who had experience single left temporoparietal CVA three years ago. Following an assessment, she gained 36 and 14 scores for receptive and expressive indexes of the MAST, respectively. Additionally, the result of the Nilipour naming test indicated that she had a severe naming deficit. The most notable feature of her speech was non-fluent verbal output. According to the motor speech assessment, she had speech and oral apraxia. Her semantic knowledge score was 282 out of 300.

#### 2.3. Patient 3

A 44 -year -old man who had a severe left frontotemporal ischemic CVA three years ago. He was classified as having moderate naming deficits, according to Nilipour naming test. His verbal output was full of phonological paraphasia and without any apraxia. His performance on MAST indicated 48 for receptive index and 44 for an expression index. His semantic knowledge was intact. Prior to his stroke, he worked in an airline company.

#### 2.4. Patient 4

A 45-year-old female with left embolic CVA in Broca territory two years ago. According to her performance on speech and language tests, she was diagnosed with severe Broca’s aphasia, naming deficit and apraxia of speech. After baseline assessment, she refused to continue the intervention process.

### 3. Baseline Phase

In the baseline phase, the participants came to the clinic once a week for three weeks. In each session of this phase, the SLP asked the participant to name the 45 training pictures without any video or clinician modeling to establish the stability of the verbal output. These pictures (only single word) matched based on syllable complexity, phonological similarity, and frequency in Persian [15, 16]. Additionally, untrained 45 pictures have provided for the generalization that also matched with training stimuli and asked to name them in the second week of baseline and follow-up phases. The participants had 40 seconds to name each target picture. These pictures set were placed on 7½ by 12½-cm index cards for use during assessment and intervention. Prior to beginning the intervention phase, the examiner divided 45 training pictures into three groups: 15 items for modeling by the clinician, 15 items for other video modeling and 15 items for video self-modeling.

#### 3.1. Video Modeling

In order to create a video modeling by other, a person who was not speech pathologist collaborated with us. We asked her to name 15 items and recorded by video.

#### 3.2. Video Self-modeling

To provide video self-modeling, we used the patients themselves to name 15 cards and recorded by video. If the patients couldn’t name them, SLP modeled each target word verbally and asked the patients imitated them. Then, the video of the best verbal output of each picture selected and be used in intervention sessions. Only the face of the person naming the items (whether other or patients themselves) showed in all video records. All video recorded played on Lenovo z500 laptop 15.5inch.

#### 3.3. Clinician Modeling

The fifty items for clinician modeling did not have any video and modeled by SLP in the intervention sessions. The patient sat in front of the clinician at a distance of approximately 75 to 90 cm with a table in between.

### 4. Intervention Phase

After providing all video of target words, the intervention phase started. Whereas high intense interventions are more effective [17], we used intense principle in our study. Thus, nine sessions of 150 minutes per day were provided for three weeks (total of 22.5 hours). In each intervention session, all three group items practiced and we consumed 50 minutes for each group. For video modeling by other and video self-modeling, the SLP placed a picture card in front of the patients and simultaneously played video recording and repeated two times. For clinician modeling after presents picture card, SLP modeled the target two times and then pressed the next item. Finally, after each three intervention sessions (the end of each week), one assessment session administrated.

### 5. Follow-up Phase

Then, patients went into the follow-up phase for 3 weeks, one day a week. To assess the effect of the intervention, we used the same 45 training target pictures during all the assessment sessions.

### 6. Outcomes Assessment

All the patients’ responds score were recorded by the first author, whereby scores of 0= no respond, 1=response with paraphasia, and 2 = correct response. Also, we used the timer app in order to score the speed of the patients’ responses. It must be pointed out that all assessment and intervention sessions were video recorded.

## Results

In this study, we compared the effects of video modeling, self-modeling, and clinician modeling on naming skills in aphasic patients. Also, we tried to find out the effect of different types of modeling on reaction time. After the baseline phase, one of the patients (no. 4) refused to continuous study and finally three patients complete intervention protocol

### Patient 1

The score of the naming skill for clinician modeling at the baseline was1.33±1.15. This score was improved to 18±4 in the intervention and reached to 16.66±3.87 in follow-up phases. The score of naming skill for video modeling by other increased from 0.66±1.15 at the baseline to 17.33±1.15 in the treatment phase and then decreased to 15.66±2.08 during follow-up phase (Table-1).

### Patient 2

In the baseline phase, the score of naming skill by clinician modeling was 0.66±1.15 that showed an increase during the treatment phase and reached to 7.66±2.08 in the follow-up phase. In the section of video modeling by other, the score of naming skill increased from pre-treatment to following the treatment.

Regarding video self-modeling, the increased score from 0.66±1.15 to 10.66±2.51 can be contributed to intervention and decreased to 8.66±3.055 during the follow-up phase (Table-2).

### Patient 3

The naming score of items modeled by clinician improved from bassline to 18.66±0.57 during follow-up phase.

In the baseline phase, the score of naming skill through video modeling by other was indicated increase during follow-up.

The scores of video self-modeling at the baseline phase was 13±3 that improved following intervention phase and reached to 17±1 during follow-up ( Table-3).

As shown in Table-4, all types of modeling could decrease time latency for naming items. Indeed, video-self modeling was the most effective way to improve the time response for all three patients.

## Discussion

For the first time, in this pilot study, we have addressed not only the efficacy of clinician modeling and video- modeling by others, but also video self-modeling on naming skills in Iranian aphasic patients. The present study revealed that the video-based approaches, as well as clinicians modeling, were effective in improving naming deficit of patients with aphasia.In this study, the positive effects of video-based modeling on naming skills of aphasic patients are consistent with other studies, which have shown the video-based modeling is effective for teaching new skills or behaviors [3-6, 8, 18-22]. Additionally, our finding shows that the video-based approaches were most effective intervention method in acquiring language disorder population that also coincides with the results of previous studies [23, 24]. The possible explanation for improved naming scores could be due to selective focus on relevant stimuli and reduce distraction and increase learning [25]. Also, the capacity of repetition video records helps to establish and maintain the behavior in memory, thus it could increase the chance of retention intervention even during the follow-up phase. Our finding is consistent with the report of Kurland *et al*. that showed repetition as a neuroplasticity principle is an important factor for learning even during chronic aphasia [26].Responding to a device rather than to clinician accompanied by less stress and pressure on patients and improves their functions [11]. The previous studies about comparisons of video-based instruction procedures involving the use of ‘self’ and ‘other’ as a model indicated no differences regarding their effectiveness, similarly, the finding of our study, showed no significant difference approximately among all three types of modeling [27-29].The results of the reaction time section have shown that the use of video-based approaches and traditional approach (clinician modeling) have an effect on patient’s processing capacity and reduced wait time for present response in patients. Although, the significance of this finding is not clear, among the three types of modeling, video self- modeling was more effective than others. Although, reduced consuming time and does not require extensive staff training prior to implementation of this type of modeling, few publications are available in the literature that discusses the efficacy of video modeling on the aphasic population.

## Conclusion

Our study indicated that video modeling by other and video self-modeling, as well as clinician modeling, are the effective ways to address naming deficits and increase the readiness to response state of chronic aphasia patients. Clearly, further research will be required to prove other issues.

## Acknowledgment

The authors would like to thanks the assistance and support of Elham Esmaili and Marzieh Asghari. This research was supported by the Semnan University of Medical Sciences (grant No. A-10-333-6).

## Conflict of Interest

All the authors report no conflicts of interest.

**Table 1:** The Naming Scores of Patient 1

		**Baseline phase**			**Intervention phase**			**Follow-up phase**		
		**S1**	**S2**	**S3**	**S4**	**S5**	**S6**	**S7**	**S8**	**S9**
**Clinician modeling**	**Score**	0	2	2	22	18	14	21	15	14
	Mean±SD	1.33±1.15	18±4	16.66±3.87
**Video modeling**	Score	0	2	0	16	18	18	18	14	15
	Mean±SD	0.66±1.15	17.33±1.15	15.66±2.08
**Video self- modeling**	Score	0	0	1	18	21	18	17	17	10
	Mean±SD	0.33±0.57	19±1.73	14.66±4.04

**S:** sessions

**Table 2:** The Naming Scores of Patient 2

		**Baseline phase**			**Intervention phase**			**Follow-up phase**		
		S1	S2	S3	S1	S2	S3	S1	S2	S3
**Clinician modeling**	Score	2	0	0	12	12	10	10	6	7
	Mean±SD	0.66±1.15	11.3±1.15	7.66±2.08
**Video modeling**	Score	0	0	0	8	8	13	8	5	4
	Mean±SD	0	9.66±2.88	5.66±2.08
**Video self- modeling**	Score	2	0	0	8	11	13	12	8	6
	Mean±SD	0.66±1.15	10.66±2.51	8.66±3.055

**S:** sessions

**Table 3:** The Naming Scores of Patient 3

	**Baseline phase**			**Intervention phase**			**Follow-up phase**		
	**S1**	**S2**	**S3**	**S1**	**S2**	**S3**	**S1**	**S2**	**S3**
**Clinician modeling**	Score	18	16	17	23	21	19	18	19	19
	Mean±SD	17±1	21±2	18.66±0.57
**Video modeling**	Score	16	19	19	21	23	26	23	22	23
	Mean±SD	18± 1.73	23.33± 2.51	22.66± 0.57
**Video self- modeling**	Score	10	13	16	12	20	20	16	18	17
	Mean±SD	13±3	17.33±4.61	17±1

**S:** sessions

**Table 4:** The Reaction Time. Data Are Presented as Seconds

	**Baseline**			**Intervention**			**Follow-up**		
	**CM**	** VM**	**VSM**	**CM**	**VM**	**VSM**	**CM**	**VM**	**VSM**
**Patient 1**	587.66	591	590.33	372.66	455.66	362.33	352	384.66	353
**Patient 2**	589.33	600	587	434.33	420	370.33	506.33	451.33	504.66
**Patient 3**	126.66	92.33	252.33	73.33	19.66	140.66	75.33	19	138

**CM:** Clinician modeling; **VM:** Video modeling; **VSM:** Video self- modeling
